# Comment on ‘Association Between Handgrip Strength and Cardiovascular Disease Risk in MASLD: A Prospective Study From UK Biobank’ by Lim et al.

**DOI:** 10.1002/jcsm.13854

**Published:** 2025-06-04

**Authors:** Mingyuan Zhao, Jiang Zhao

**Affiliations:** ^1^ Department of Oncology Shanxi Provincial People's Hospital, Affiliated to Shanxi Medical University Taiyuan China; ^2^ Department of Hepatology Shanxi Provincial People's Hospital., Affiliated to Shanxi Medical University Taiyuan China

We recently read an article by Lim TS et al. [1] published in the Journal of Cachexia, Sarcopenia and Muscle entitled ‘Association Between Handgrip Strength and Cardiovascular Disease Risk in MASLD: A Prospective Study From UK Biobank.’ This study focused on an important health issue in the metabolic dysfunction‐associated steatotic liver disease (MASLD) population and attempted to predict cardiovascular disease (CVD) risk using a simple and feasible handgrip strength (HGS) test. However, we have also noticed some potential limitations that may affect the interpretation and generalization of the research results.

Firstly, in the abstract [1], ‘The incidence of CVD events increased as HGS decreased in participants with MASLD, regardless of the presence or absence of advanced liver fibrosis’. However, advanced liver fibrosis was defined by a fibrosis‐4 (FIB‐4) score > 2.67. While the FIB‐4 is convenient and easy to use, its limitations need to be recognized, especially in specific populations such as patients with Type 2 diabetes mellitus (T2DM) [2]. The AGA recommends a second‐tier noninvasive test, such as vibration‐controlled transient elastography, for all T2DM patients, regardless of FIB‐4 results, differing from other organizations [3]. The EASL‐EASD‐EASO guidelines has never mentioned that when the FIB‐4 score > 2.67, it was a method for screening liver fibrosis in patients with T2DM [4]. MASLD is a continuous spectrum, the progressing from simple steatosis to metabolic‐associated steatohepatitis, and may eventually lead to liver fibrosis, liver cirrhosis, liver failure and hepatocellular carcinoma (HCC) [4]. Different stages of MASLD have different metabolic and inflammatory states, and these differences may affect the CVD [5, 6]. MASLD has a high degree of heterogeneity, including different etiologies, severity of disease and comorbidities. This heterogeneity may be the true relationship between HGS and CVD risk. More refined stratification among MASLD patients is needed, such as by degree of inflammation, severity of steatosis and of T2DM, to evaluate the association of HGS with CVD events separately.

Secondly, Lim TS et al. pointed out that ‘the importance of investigating whether interventions for enhancing muscle strength could effectively decrease CVD risk in patients with MASLD.’ This indicates that the causal relationship between HGS and the CVD risk had become a foregone conclusion. Although it is a prospective study, the observational nature of the study makes it difficult to determine the exact causal relationship between HGS and the CVD risk in patients with MASLD. There may be other unmeasured or undiscovered confounding factors that simultaneously affect HGS, MASLD and CVDs, leading to misjudgment of the relationship among them. Longitudinal studies with repeated assessments of HGS, MASLD and CVDs over an extended period could shed light on the trajectories of these variables and their interplay over time. To make inferences about causality, Mendelian randomization studies can also be used [7].

Additionally, this study utilized data from UK Biobank to investigate the relationship between HGS and the CVD risk in patients with MASLD. Although prospective, the factors such as liver disease progression and changes in important variables may have an impact on the research results during the 13.1 years follow‐up period [5, 6]. Therefore, these limitations should be emphasized more strongly in the paper, and their potential impact on the research results should be discussed.

Lastly, the findings from this study need to be validated in other populations. The UK Biobank recruited primarily individuals of British residents, so the findings may not apply to other racial or cultural groups. In addition, validation in different clinical settings, such as in community populations or high‐risk groups, is also needed.

In conclusion, although the study by Lim et al. has provided valuable information for us to understand the relationship between HGS and the risk of CVD in patients with MASLD, we also need to recognize its limitations. Future studies should be improved in the following aspects: more precise diagnosis of MASLD, stricter control of confounding factors, verification of causal relationships and expansion of the study population.

## Conflicts of Interest

The authors declare no conflicts of interest.

## References

[1] T. S. Lim , S. Kwon , S. A. Bae , et al., “Association Between Handgrip Strength and Cardiovascular Disease Risk in MASLD: A Prospective Study From UK Biobank,” Journal of Cachexia, Sarcopenia and Muscle 16, no. 2 (2025): e13757. 40035094 10.1002/jcsm.13757 PMC11876860

[2] J. Kim , T. Ito , T. Arai , et al., “Modified FIB‐4 Index in Type 2 Diabetes Mellitus With Steatosis: A Non‐Linear Predictive Model for Advanced Hepatic Fibrosis,” Diagnostics (Basel) 14, no. 22 (2024): 2500. 39594165 10.3390/diagnostics14222500 PMC11592587

[3] K. Patel and G. Sebastiani , “Limitations of Non‐Invasive Tests for Assessment of Liver Fibrosis,” JHEP Reports 2, no. 2 (2020): 100067. 32118201 10.1016/j.jhepr.2020.100067 PMC7047178

[4] European Association for the Study of the Liver (EASL) , European Association for the Study of Diabetes (EASD) , and European Association for the Study of Obesity (EASO) , “EASL‐EASD‐EASO Clinical Practice Guidelines on the Management of Metabolic Dysfunction‐Associated Steatotic Liver Disease (MASLD),” Journal of Hepatology 81, no. 3 (2024): 492–542. 38851997 10.1016/j.jhep.2024.04.031

[5] G. Targher , C. D. Byrne , and H. Tilg , “MASLD: A Systemic Metabolic Disorder With Cardiovascular and Malignant Complications,” Gut 73, no. 4 (2024): 691–702. 38228377 10.1136/gutjnl-2023-330595

[6] M. Kokkorakis , E. Muzurović , Š. Volčanšek , et al., “Steatotic Liver Disease: Pathophysiology and Emerging Pharmacotherapies,” Pharmacological Reviews 76, no. 3 (2024): 454–499. 38697855 10.1124/pharmrev.123.001087

[7] C. Zhuo , J. Zhao , and Q. Wang , “Assessment of Causal Associations Between Handgrip Strength and Cardiovascular Diseases: A Two Sample Mendelian Randomization Study,” Frontiers in Cardiovascular Medicine 9 (2022): 930077. 35990959 10.3389/fcvm.2022.930077 PMC9386423

